# Supplementary material for: A-type FHFs mediate resurgent currents through TTX-resistant voltage-gated sodium channels
Source: eLife. 2022 Apr 20;11:e77558. doi: 10.7554/eLife.77558 (PMC9071269; doi:10.7554/eLife.77558)
Supplement: Transparent reporting form [file elife-77558-transrepform1.docx]

***eLife’s* transparent reporting form**

We encourage authors to provide detailed information *within their submission* to facilitate the interpretation and replication of experiments. Authors can upload supporting documentation to indicate the use of appropriate reporting guidelines for health-related research (see [EQUATOR Network](http://www.equator-network.org/%20)), life science research (see the [BioSharing Information Resource](https://biosharing.org/)), or the [ARRIVE guidelines](http://www.plosbiology.org/article/info:doi/10.1371/journal.pbio.1000412) for reporting work involving animal research. Where applicable, authors should refer to any relevant reporting standards documents in this form.

If you have any questions, please consult our Journal Policies and/or contact us: [editorial@elifesciences.org](mailto:editorial@elifesciences.org).

**Sample-size estimation**

- You should state whether an appropriate sample size was computed when the study was being designed
- You should state the statistical method of sample size computation and any required assumptions
- If no explicit power analysis was used, you should describe how you decided what sample (replicate) size (number) to use

Please outline where this information can be found within the submission (e.g., sections or figure legends), or explain why this information doesn’t apply to your submission:

The experiments here are designed to determine if A-type FHFs mediate resurgent sodium currents in heterologous expression systems and if the resurgent currents influence neuron excitability. The appropriate sample size is not computed in advance and explicit power analysis is not used either because the results are relatively consistent between samples (conditions).

**Replicates**

- You should report how often each experiment was performed
- You should include a definition of biological versus technical replication
- The data obtained should be provided and sufficient information should be provided to indicate the number of independent biological and/or technical replicates
- If you encountered any outliers, you should describe how these were handled
- Criteria for exclusion/inclusion of data should be clearly stated
- High-throughput sequence data should be uploaded before submission, with a private link for reviewers provided (these are available from both GEO and ArrayExpress)

Please outline where this information can be found within the submission (e.g., sections or figure legends), or explain why this information doesn’t apply to your submission:

The numbers of independent cells used to collect the data under control or experimental conditions are reported in Figures and Tables. All data presented in this manuscript reflect biological replicates. The voltage-clamp or current-clamp protocols are repeatedly applied on the same cells to ensure that the properties of sodium currents and neuron excitability did not change during prolonged whole-cell recordings (Technical replicates). However, the technical replicates are not presented in the manuscript. The information has been provided in Materials and Methods (lines 429-475 and lines 491-495). No outliers are encountered.

**Statistical reporting**

- Statistical analysis methods should be described and justified
- Raw data should be presented in figures whenever informative to do so (typically when N per group is less than 10)
- For each experiment, you should identify the statistical tests used, exact values of N, definitions of center, methods of multiple test correction, and dispersion and precision measures (e.g., mean, median, SD, SEM, confidence intervals; and, for the major substantive results, a measure of effect size (e.g., Pearson's r, Cohen's d)
- Report exact p-values wherever possible alongside the summary statistics and 95% confidence intervals. These should be reported for all key questions and not only when the p-value is less than 0.05.

Please outline where this information can be found within the submission (e.g., sections or figure legends), or explain why this information doesn’t apply to your submission:

The approaches of statistical analysis are presented in the section Materials and Methods (lines 491-495) and in figure legends. χ^2^ analysis is performed in figure 5g (also line 191 in MS) and figure 6j (also line 217), respectively. One-way ANOVA analysis is performed in figure 6b and c. If not indicated, Student’s t-test is performed. The numbers and exact p-values are reported in figure legends and in Results. Figures show means and standard errors.

(For large datasets, or papers with a very large number of statistical tests, you may upload a single table file with tests, Ns, etc., with reference to sections in the manuscript.)

**Group allocation**

- Indicate how samples were allocated into experimental groups (in the case of clinical studies, please specify allocation to treatment method); if randomization was used, please also state if restricted randomization was applied
- Indicate if masking was used during group allocation, data collection and/or data analysis

Please outline where this information can be found within the submission (e.g., sections or figure legends), or explain why this information doesn’t apply to your submission:

In our studies, the acquisition of control and experimental data is randomized. This information has been provided in the section Materials and Methods (lines 491-492). The cells transfected with scramble or FHF4shRNA are allocated into control or experimental groups, respectively. The FHF4shRNA-transfected DRG neurons without or with F4A peptide are allocated into control or experimental groups, respectively. The DRG neurons allocated into control or experimental groups are isolated from the same rat. Masking is not necessary.

**Additional data files (“source data”)**

- We encourage you to upload relevant additional data files, such as numerical data that are represented as a graph in a figure, or as a summary table
- Where provided, these should be in the most useful format, and they can be uploaded as “Source data” files linked to a main figure or table
- Include model definition files including the full list of parameters used
- Include code used for data analysis (e.g., R, MatLab)
- Avoid stating that data files are “available upon request”

Please indicate the figures or tables for which source data files have been provided:

All available data has been represented in the manuscript.
